# Serum matrix metalloproteinase-9 in colorectal cancer family-risk population screening

**DOI:** 10.1038/srep13030

**Published:** 2015-08-12

**Authors:** Olalla Otero-Estévez, Loretta De Chiara, Mar Rodríguez-Girondo, Francisco Javier Rodríguez-Berrocal, Joaquín Cubiella, Inés Castro, Vicent Hernández, Vicenta Soledad Martínez-Zorzano

**Affiliations:** 1 Department of Biochemistry, Genetics and Immunology, Universidade de Vigo. Vigo, Spain; 2 SiDOR Research Group & Centro de Investigaciones Biomédicas (CINBIO), Universidade de Vigo. Vigo, Spain; 3 Department of Gastroenterology, Complexo Hospitalario Universitario de Ourense. Ourense, Spain; 4 Department of Gastroenterology, Complexo Hospitalario Universitario de Vigo. Vigo, Spain

## Abstract

Matrix metalloproteinase-9 (MMP-9) is related to tumour development and progression in colorectal cancer (CRC) and its utility as biomarker has been suggested. The aim of our study was to measure serum MMP-9 in asymptomatic first-degree relatives of CRC patients, and to analyse its diagnostic accuracy for the detection of advanced neoplasia (AN: advanced adenomas and CRC). Additionally, we compared its diagnostic capability with the most used non-invasive faecal immunochemical test (FIT). Serum MMP-9 was quantified by ELISA in 516 asymptomatic individuals that underwent a colonoscopy and a FIT. MMP-9 levels were significantly related to age and gender and therefore the concentration was corrected by these confounders. Corrected MMP-9 (cMMP-9) levels were higher in individuals with advanced adenomas (AA; *p*-value = 0.029) and AN (*p*-value = 0.056) compared to individuals with no neoplasia. Moreover, elevated cMMP-9 concentration was associated with more severe characteristics of adenomas (number of lesions, size and histology). Nevertheless, the diagnostic accuracy of cMMP-9 was considerably lower than that of FIT for identifying AA (22.64% vs. 47.17% sensitivity, 90% specificity) or AN (19.30% vs. 52.63% sensitivity, 90% specificity). According to our results, serum MMP-9 cannot be considered of utility for the diagnosis of AN in CRC family-risk population screening.

Colorectal cancer (CRC) is the third most common cancer worldwide, responsible for more than 600000 deaths every year. When the disease is detected at stage I, patients have a 5-year survival rate of more than 90%, while individuals diagnosed at stage IV only reach 10%^1^. Thus, the early detection of cancer as well as the removal of precancerous adenomas is essential to achieve improvements in survival rates.

Individuals with first-degree relatives (FDRs) with CRC have a 2 to 4-fold higher risk of developing this cancer^2^, together with an increased-risk of presenting advanced adenomas (AA)^3^. The most accurate but invasive detection technique for tumours and adenomas is the colonoscopy, with an adherence lower than 43% in this population^4^,^5^. On the other hand, nowadays the most valuable non-invasive screening option is the faecal immunochemical test (FIT), which shows high sensitivity for the detection of CRC but inadequate performance for AA^6^,^7^. Therefore, the development of new non-invasive screening strategies for the detection of advanced neoplasia (AN: CRC and AA) in these individuals at increased-risk is of great importance.

Matrix metalloproteinases (MMPs) are zinc-dependent endopeptidases that have an important role in the degradation of extracellular matrix components, crucial for tumour growth, invasion and metastasis^8^,^9^. This large family of proteins also has shown modulating functions in immunity and inflammation during tumorigenesis through the activation of non-matrix substrates including growth factors, cytokines and other membrane proteins^10^,^11^,^12^.

Increased serum concentrations of certain MMPs have been described in different types of cancer, suggesting their utility as biomarkers^9^,^10^. In CRC, enhanced expression of several MMPs (MMP-1, −2, −7, −9 and −13) has been reported. The type IV collagenase MMP-9 (gelatinase B; EC 3.4.24.35) is of special interest in the progression of this neoplasia, and might also play a role in the colorectal carcinogenesis from adenoma to carcinoma^8^,^13^. MMP-9 is involved in angiogenesis by regulating the bioavailability of the pro-angiogenic vascular endothelial growth factor^8^,^14^. This protein is expressed and secreted as an inactive form named pro-MMP-9 (92 kDa), which is then activated by proteolysis of the propeptide domain resulting in an 82 kDa form. Moreover, a reversible activation can be initiated with the binding to type IV collagen^11^,^15^.

Immunohistochemical studies demonstrated an over-expression of MMP-9 in colorectal tumours compared with healthy mucosa^16^,^17^. This up-regulation of MMP-9 has also been described in precursor lesions suggesting its utility as a biomarker for early diagnosis of CRC^18^,^19^,^20^. Accordingly, a correlation between tumour MMP-9 expression and preoperative serum levels of MMP-9 has been observed in individuals with CRC^17^. Elevated serum MMP-9 levels were previously reported in case-control studies of CRC patients and healthy subjects^21^,^22^, and in symptomatic cohorts including patients with advanced neoplasia^23^,^24^. However, the diagnostic utility of serum MMP-9 is still uncertain since some authors suggest a possible predictive value for the detection of AN^23^,^24^, while others report little or no diagnostic capability^21^,^25^,^26^.

Currently, there is no evidence of the utility of serum MMP-9 for the diagnosis of CRC in asymptomatic individuals with a family history of CRC. Therefore, the aim of our study was to measure serum levels of MMP-9 in asymptomatic FDRs of CRC patients, and to analyse its diagnostic performance for the detection of AN. In addition, we compared the diagnostic accuracy of this marker with the most used non-invasive FIT.

## Results

### Colonoscopy and FIT in the study population

The study cohort included 516 FDRs of CRC patients (212 men and 304 women). The age of all participants ranged from 28 to 84 years (median: 54.21 years). According to the colonoscopy findings individuals were classified as: 174 (33.72%) with no colorectal findings, 164 (31.78%) with benign pathologies [haemorrhoids (n = 68), diverticula (n = 46), no-neoplastic polyps (n = 44), and other minor findings (n = 6)], 121 (23.45%) cases of non-advanced adenomas (NAA), 53 (10.27%) individuals with AA and 4 CRC cases (0.78%).

The faecal haemoglobin concentration ranged between 0-3739 ng/mL, with a noticeable skewed distribution due to the total absence of blood (0 ng haemoglobin/mL) in 275 individuals (53.3% of the cohort). Additionally, the presence of extreme values in some cases resulted in a high variance (83583). As specified in the Methods section, we performed a logarithmic transformation of the FIT data [log_10_(FIT + 2)] to reduce the skewness. After transformation, the variance was reduced to 0.42, and the range was also diminished to 0.30–3.57 ng/mL compared to the untransformed data.

The comparison of the transformed haemoglobin concentration [log_10_(FIT + 2)] resulted in statistically significant differences when the groups no neoplasia, NAA, AA and CRC were compared (Kruskal-Wallis test, *p*-value < 0.001), as well as the comparison between AN and the rest of the groups (Mann-Whitney U test, *p*-values < 0.001; data not shown).

### MMP-9 levels and demographic characteristics of the cohort

Serum MMP-9 levels varied from 31 to 2338 ng/mL, and the median concentration for the entire cohort was 410 ng/mL. MMP-9 levels were analysed according to the demographic and clinical characteristics of individuals (Table 1). We found statistically significant differences in MMP-9 concentration regarding age (Kruskal-Wallis test, *p-*value < 0.001), with increased levels in individuals aged ≤49 years followed by individuals ≥60 years [median difference −49 ng/mL, 95% bootstrap CI: −114–(−4)], while individuals with 50–59 years showed the lowest MMP-9 concentrations [median difference −75 ng/mL, 95% bootstrap CI: −138–(−34)]. In relation to gender, men exhibited slightlty increased MMP-9 levels compared to women (median difference −20 ng/mL, 95% bootstrap CI: −86–17; Mann-Whitney U test, *p*-value = 0.013). On the other hand, no differences were observed for the familial risk in relation to the number or age of the relatives with CRC (Mann-Whitney U test, *p*-value = 0.652).

Age and gender distributions differed according to the colonoscopy findings of the patients analysed in our cohort. Namely, most of the advanced neoplasias corresponded to men and the age groups 50–59 and ≥60, while no neoplasia was constituted largely by women and the youngest age group (see Supplementary Table S1 online).

### MMP-9 levels according to the colorectal findings

**Figure 1:**
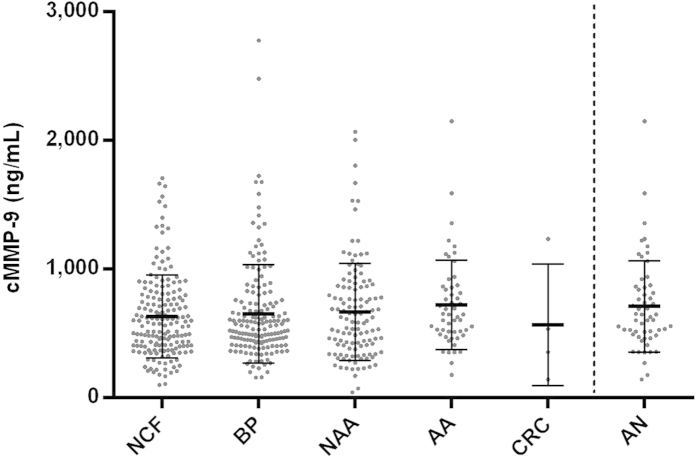
Scattered plot of the serum MMP-9 levels corrected by age and gender (cMMP-9) according to the pathological groups. NCF: no colorectal findings; BP: benign pathologies; NAA: non-advanced adenomas; AA: advanced adenomas; CRC: colorectal cancer; AN: advanced neoplasia. Middle lines represent the mean cMMP-9, while whiskers represent the standard deviation.

The crude concentration of serum MMP-9 is presented online on Supplementary Table S2 for all the individuals analysed based on the colonoscopy result. The no neoplasia group showed mean and median levels lower than the AN group (463 ± 264 ng/mL; 398 ng/mL vs. 515 ± 281 ng/mL; 427 ng/mL). However, as we found that the concentration of MMP-9 is related to age and gender of the individual, in order to perform an accurate analysis in relation to the colorectal pathologies we corrected the MMP-9 levels for these confounders, as detailed in the Methods section. The corrected levels of serum MMP-9 (cMMP-9) according to the pathological groups is represented in Fig. 1. The results of the statistical analysis performed using the cMMP-9 is shown on Table 2.

Based on the corrected concentration, individuals with no colorectal findings (633 ± 322 ng/mL) and benign pathologies (653 ± 381 ng/mL) showed comparable cMMP-9 levels, with no statistically significant differences (Mann-Whitney U test, *p*-value = 0.997). Differences were also absent between no colorectal findings group and each of the sub-groups included as benign pathologies (Mann-Whitney U tests, *p*-values > 0.05). Individuals diagnosed of NAA (668 ± 379 ng/mL) showed similar concentrations compared to no neoplasia (Mann-Whitney U test, *p*-value = 0.579); however, patients with AA registered higher cMMP-9 levels (722 ± 349 ng/mL), differing statistically from the no neoplasia group (Mann-Whitney U test, *p*-value = 0.029). In relation to CRC, the mean concentration found for these 4 patients (568 ± 474 ng/mL) was contrarily the lowest among the pathological groups. However, this result should be cautiously interpreted due to the reduced number of CRC patients (stage I cases: 145 ng/mL and 355 ng/mL; stage II case: 1237 ng/mL; stage III case: 537 ng/mL). Since the aim of a screening programme is the detection of CRC and AA, results are presented for the AN group (712 ± 356 ng/mL), showing statistical differences near significance when compared to no neoplasia (median difference 66 ng/mL, 95% bootstrap CI: −31–190; Mann-Whitney U test, *p*-value = 0.056).

### Relationship between cMMP-9 and the histopathological characteristics of adenomas

The corrected MMP-9 levels were also studied regarding the characteristics of adenomas (number of lesions per individual, size, histology and location; Table 3). The most severe characteristics of adenomas that could be analysed in our study (3 or more lesions, size ≥1 cm, tubulovillous or villous histology) showed higher cMMP-9 values compared to the mild characteristics, though no statistical significant differences were reported for any of the features. Consequently and according to the definition of AA, this tendency for higher cMMP-9 levels was also observed for these premalignant lesions compared to NAA (see Table 2) though no statistical significant differences were found (Mann-Whitney U test, *p*-value = 0.176). In relation to the location of the adenomas, we found similar cMMP-9 levels for distal, proximal or both locations, indicating no particular trend.

### Diagnostic performance of MMP-9 and FIT

**Figure 2:**
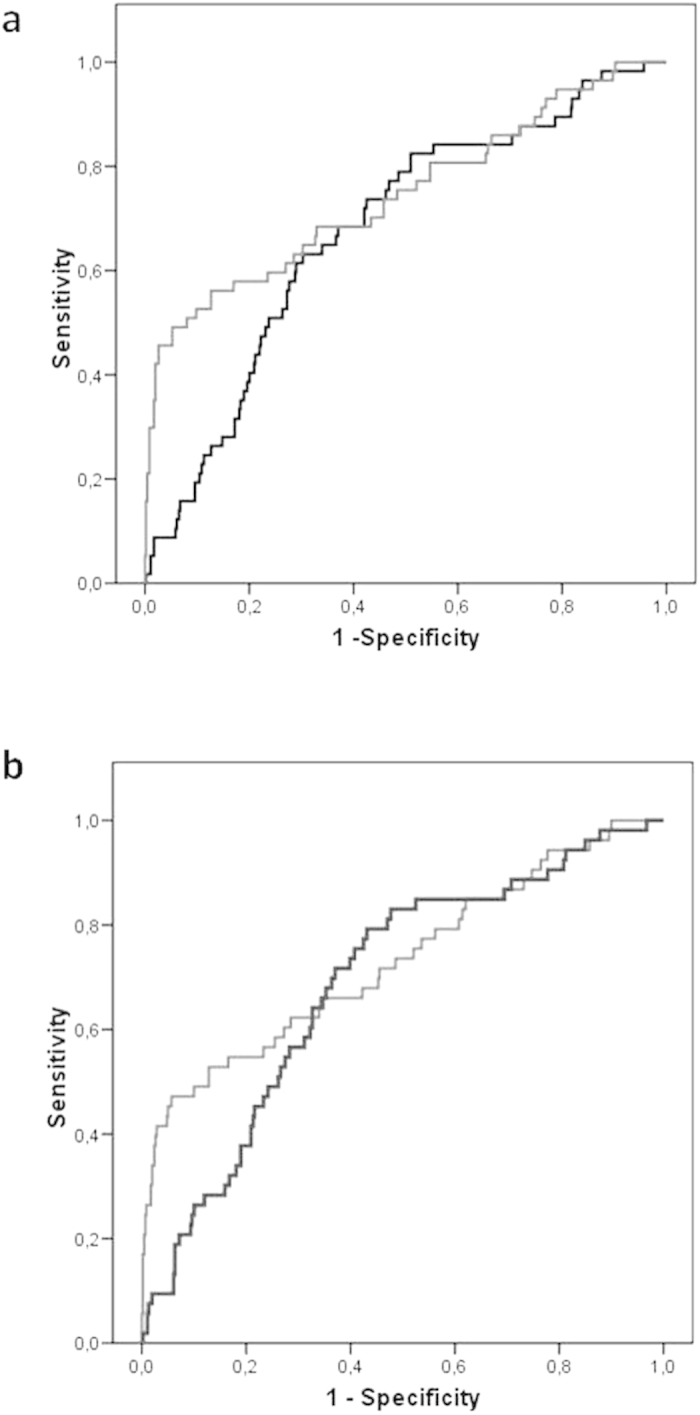
ROC curves for the diagnosis of advanced neoplasia (A) and advanced adenomas (B). The black curves corresponds to serum MMP-9 (log_10_MMP-9) corrected by confounders gender and age; the grey curves corresponds to FIT [log_10_(FIT + 2)] corrected by confounders gender and age.

To evaluate the diagnostic performance of MMP-9 and FIT as biomarkers, ROC curves were generated on one hand for the detection of AN, and on the other for AA (Fig. 2A,B, respectively). Since gender and age are factors that were found to be associated with the presence of advanced neoplasias, the diagnostic capability of the markers were analysed using logistic regression models that included gender and age to adjust for these confounding variables, as described in the Methods section. The area under the curve (AUC) for MMP-9 (log_10_MMP-9) was 0.678 (95% CI: 0.635–0.718) for the detection of AN, while for FIT [log_10_(FIT + 2)] this resulted superior (0.744, 95% CI: 0.704–0.781). The difference between these ROC curves resulted in 0.066, which is significantly positive according to the boostrap method (95% CI: 0.009–0.135), almost reaching statistical significance (DeLong test, *p*-value = 0.0588).When specificity was set to 90% or 95% the faecal test detected 52.63% and 45.61% of the AN lesions, respectively, compared to a reduced 19.30% and 8.77% sensitivity observed for MMP-9 (Table 4).

The analysis of the diagnostic performance of the markers for detecting AA excluded the 4 CRC cases. The ROC curve for MMP-9 (log_10_MMP-9) showed an AUC of 0.689 (95% CI: 0.647–0.729), resulting in a sensitivity a bit superior to that for detecting AN, but likewise inadequate for a screening test. In the case of FIT [log_10_(FIT + 2)], the AUC was 0.731 (95% CI: 0.690–0.769), which was not statistically different from that with MMP-9 (DeLong test, *p*-value = 0.208), with a difference between AUCs of 0.042 (boostrap method 95% CI: −0.015–0.104). However, the estimated power for this last comparison is considerably low, around 20% following the method described in Obuchowski & McClish (1997)^27^, so the result should be cautiously interpreted. As in the case of AN, FIT demonstrated for both specificities reported on Table 4, a superior sensitivity for detecting AA compared to MMP-9.

## Discussion

Many of the studies conducted up to date on the MMP-9 in CRC are focused on the correlation of its expression in tissue and the clinicopathological features of the tumour, besides the association with its concentration or enzymatic activity measured in serum, suggesting its use as a prognostic factor^28^,^29^,^30^. In relation to diagnosis, results are variable and inconclusive^21^,^23^,^24^,^25^,^26^.

In order to further evaluate this molecule we analysed the levels of serum MMP-9 in relation to the colonoscopy findings in a family-risk cohort including asymptomatic individuals with the aim of determining its diagnostic utility for the detection of AN. To our knowledge this is the first study that measures serum MMP-9 in a cohort of these characteristics, and additionally compares the performance of this molecule with that of FIT.

Serum MMP-9 measurements available in literature derive mainly from case-control studies and cohorts including symptomatic individuals. Mroczko *et al.* (2010)^21^, Wilson *et al.* (2012)^24^ and Damery *et al.* (2013)^25^ described for individuals with no neoplasia crude median MMP-9 serum concentrations of 254–361 ng/mL, inferior to the 398 ng/mL median from our cohort for this group. In the case of individuals with advanced neoplasia, the levels reported by the above mentioned studies ranged from 380 to 530 ng/mL, more alike with the 427 ng/mL median concentration we found for the AN group. The difference among individuals with no neoplasia may be related to the criteria used to define this ‘control/healthy’ group (abdominal ultrasound or computed tomography examination^21^, not colonoscopy as in our study), or the presence of symptoms unlike our cohort^24^,^25^.

In our cohort we found that the younger group of individuals (≤49 years) showed higher crude MMP-9 levels compared to the 50–59 and ≥60 years groups, resulting in statistically significant differences, as well as gender, with men showing higher concentrations than women. Similarly, Hurst *et al.* (2007)^23^ reported for disease-free controls reduced MMP-9 serum concentrations with advancing age, though no further details were provided. The variations in the MMP-9 serum levels intrinsic to age and gender described in our family-risk cohort, together with the epidemiological fact that both AA and CRC have a higher prevalence in males and in older-aged groups and hence are factors considered predictive of colorectal neoplasia^1^,^31^, well justify the need of correcting the concentration of this metalloproteinase by these confounders. This correction allowed us to make an appropriate statistical analysis to determine differences in MMP-9 related to colorectal pathologies diagnosed through colonoscopy and according to the histopathological characteristics of adenomas, ruling out that differences are due to the age or gender of the individual.

In our study we found an increase in the corrected median MMP-9 serum concentration from individuals with no neoplasia—non-advanced adenomas—advanced adenomas (564 ng/mL–599 ng/mL–648 ng/mL). As a result, statistically significant differences were detected when we compared cMMP-9 levels in no neoplasia and AA (*p*-value = 0.029), suggesting a possible diagnostic utility.

In relation to cancer, the expected elevation of this molecule was not evidenced. Regardless of the reduced number of neoplasia cases, we found that corrected MMP-9 levels were increased in stage II and III patients compared to stage I patients. Such differences in the expression of MMP-9 among tumour stages have also been described for tumour tissues (higher staining scores)^20^,^30^. In the case of serum samples, Biasi *et al.* (2012)^29^ reported increased MMP-9 enzymatic activity (zymography) and protein levels (ELISA) only for stage II and III cancers, comparable to the tendency we found. They also confirmed by immunohistochemistry that MMP-9 was clearly produced in large amount in tumours at stage II and III.

Besides cancer, the goal of a screening initiative also includes the detection of AA. When these pathologies were grouped as AN, the difference in the corrected MMP-9 levels compared to no neoplasia became near significance (*p*-value = 0.056). Hurst *et al.* (2007)^23^ demonstrated statistically significant differences in the expected (age-specific predicted) MMP-9 values between no neoplasia and neoplastic conditions, as well as Mroczko *et al.* (2010)^21^ and finally Wilson *et al.* (2012)^24^ based on the variable MMP-9 quartiles. Unlike the previous studies, the group headed by Dr Ismail in their latest work did not find an association between serum MMP-9 levels and neoplasia (high-risk polyps and cancer) in symptomatic patients referred from primary care to a secondary care colorectal clinic^25^.

The inclusion of all the diagnostic groups recruited in a screening setting constitute one of the strengths of our study, and has allowed us to perform a complete analysis regarding the histopathological characteristics of adenomas, which has not been reported previously in literature. We studied the features of the 174 adenomas included in our cohort (121 non-advanced and 53 advanced lesions) and found that the most severe characteristics such as 3 or more lesions, size ≥1 cm, and tubulovillous or villous histology corresponded to higher levels of corrected MMP-9, over 700 ng/mL. In spite of this tendency for the number of adenomas, size and histology, the differences were not statistically significant for any of this features, and neither between NAA and AA. Though the presence of dysplasia is a critical characteristic considered a morphological marker for neoplastic lesions, we could not include this feature because all our adenomas showed low-grade dysplasia. The only study analysing serum MMP-9 in adenomas was limited to 28 lesions and indicated similar levels of this metalloproteinase for tubular and tubulovillous adenomas, though higher than controls^29^. In relation to the location of adenomas, serum corrected MMP-9 levels were equivalent for the distal, proximal or distal and proximal colorectal lesions included in our study. This result coincides with that from Daniel *et al.* (2007)^19^ reporting no differences in MMP-9 expression according to the location of adenomas.

Our observations about MMP-9 levels in serum samples are in agreement with the progressive increase of tissue MMP-9 in the mucosa—adenoma—carcinoma multi-step process described by several authors. Immunohistochemical studies analysing different tissue samples including normal mucosa, hyperplastic polyps, adenomas and tumours coincide in the gradual increase of the staining intensity in this sequence corresponding to the progressive expression of MMP-9^20^,^32^. In their works they have indicated more intense staining in tubulovillous and villous adenomas compared to tubular epithelia, with higher MMP-9 expression in tubulovillous adenomas with high-grade dysplasia than in tubular or tubulovillous adenomas with low-grade dysplasia^19^,^20^,^32^.

Based on the up-regulation of this metalloproteinase as an early event in the adenoma-carcinoma sequence, some studies have centred on the diagnostic utility of MMP-9. The results of our study suggested a potential diagnostic use of serum MMP-9 to detect AN, including elevated levels in individuals bearing AA, tendency of higher levels related to more severe characteristics of adenomas, and lastly, similar concentrations among no colorectal findings and the benign no-neoplastic pathological groups. However, the diagnostic sensitivity of MMP-9 to detect AN or AA was not the expected when specificity was set to 90 or 95%, indicating a poor performance and hence a lack of utility for detecting these colonic lesions, at least for the screening of an asymptomatic family-risk cohort like ours.

The studies available in literature analysing serum MMP-9 as a diagnostic test are limited. The group headed by Dr Ismail, focused on individuals bearing gastrointestinal symptoms, has performed three studies. The first two proposed a model combining certain symptoms and logMMP-9^23^ or MMP-9 quartiles^24^ for the detection of advanced neoplasia, reporting sensitivities and specificities in both cases towards 78 and 75%. However, in their last study they concluded that serum MMP-9 appears to have little value as a tool to aid referral decisions in the symptomatic population^25^. Regardless of the difference in theirs and our cohort in relation to symptoms, serum MMP-9 shows no diagnostic utility for detecting AN in any of the contexts. The suboptimal performance of MMP-9 was also evidenced by Mroczko *et al.* (2010)^21^ reporting a 55% sensitivity for CRC, and more recently by Pengjun *et al.* (2013)^26^ that do not include this metalloproteinase in their serum panel for the detection of CRC.

The inadequate diagnostic capability of serum MMP-9 seems even more evident when directly compared to the FIT, which is considered the most valuable non-invasive option. The evaluation of both tests in all the individuals participating in our study constitutes a strong point in our work, helping to support that the search for new non-invasive markers has to continue since serum MMP-9 cannot be considered of utility for the diagnosis of CRC and AA in a screening setting where at least a 90% specificity is essential and a reasonable sensitivity is desirable.

## Methods

### Study population

This is a prospective, controlled, blinded, cohort study that included asymptomatic individuals with at least one FDR with CRC consecutively referred to undergo a colonoscopy as a CRC screening method, recruited from the *Complexo Hospitalario Universitario de Ourense* from January 2010 to December 2011. Exclusion criteria comprised a personal history of CRC, adenomas or inflammatory bowel disease, hereditary CRC or a colonoscopy examination within the past 5 years. The study was approved by the Galician Ethical Committee for Clinical Research (2009/153) and complied with the tenets of the Helsinki Declaration and the Oviedo Agreement. The methods carried out in the study are in accordance with the approved relevant guidelines and regulations. All participants gave written informed consent and their anonymity was warranted.

### FIT and colonoscopy

For each individual a stool sample was obtained one week before colonoscopy. The faecal occult blood (ng of haemoglobin per mL) was measured with a quantitative immunochemichal test (OC-sensor; Eiken Chemical; Tokyo, Japan).

All these individuals underwent a colonoscopy, blind to the FIT, performed by experienced endoscopists who had done >200 colonoscopies per year, following the recommendations of the Spanish guidelines on quality of colonoscopy in colorectal cancer screening^33^. The endoscopic procedure allowed the classification of the individuals regarding the most advanced lesion. Polyps were divided into non-neoplastic (inflammatory and hyperplastic) or neoplastic (non-advanced adenomas and advanced adenomas). Advanced adenomas are defined as adenomas greater or equal to 1 cm with tubulovillous or villous histology, high-grade dysplasia or intramucosal carcinoma. Cancer stages were based on the AJCC classification^34^. Advanced neoplasia included CRC and AA. In relation to the location of adenomas, these were classified as ‘proximal’ when located only proximal to the splenic flexure of the colon, ‘distal’ when found only in the distal colon or ‘both’ when lesions were present in distal and proximal locations.

### Serum samples and MMP-9 measurement

Venipuncture was performed one week before the endoscopic procedure. Sera were obtained by centrifugation of blood at 2000 *g* for 15 min and were stored at −20 °C.

The serum MMP-9 concentration (ng/mL) was measured with the Human MMP-9 Quantikine ELISA Kit (R&D Systems, Abingdon, UK) according to the manufacturer’s instructions. As indicated in the kit, serum samples were diluted 100-fold. Colorimetric quantification was performed by duplicate with a microplate reader (model 550; Bio-Rad; Hercules, CA, USA) at 450/570 nm. MMP-9 was quantified blind to the colonoscopy and FIT results.

### Sample size calculation

Since the ultimate goal of the study is to evaluate the ability of MMP-9 to detect advanced neoplasia, we conducted a power analysis to determine the required sample size to discriminate between advanced neoplasia and no neoplasia groups. Specifically, under the assumption of binormal ROC curves, at least 43 cases of advanced neoplasia are needed to significantly detect AUCs larger than 0.65 ensuring a power of 80%^27^. Moreover, such number of cases would allow the detection of differences in sensitivities and specificities of around a 17% with the same detection power based on a McNemar test.

### Data analysis

Continuous variables are presented as mean with standard deviation, median and range. Additionally, bootstrap (based on B = 2000 resamples) confidence intervals for the differences in median among subgroups are also provided. Non-parametric statistic was used for multiple group comparisons (Kruskal-Wallis test) and for two-sample group comparisons (Mann-Whitney U test). FIT values were transformed to log_10_(FIT + 2) to reduce the skewness before performing statistical analyses. Since age and gender were associated with both the serum MMP-9 levels and colorectal findings, a corrected value of MMP-9 was derived to avoid the masking of the MMP-9 effects by confounding with these variables. Namely, a new variable, cMMP-9, was obtained by extracting the amount of variation in the levels of MMP-9 attributable to age and gender in the ‘healthy’ population (no colorrectal findings group). This was estimated by fitting a linear regression model with log_10_(MMP-9) as outcome and age and gender as regressors in the subsample without colorectal findings.

The ability of FIT and MMP-9 to separate individuals with no neoplasia from those with AN or AA was studied in terms of receiver-operating characteristic (ROC) curves. To account for the different impact of age and gender in each of the markers, we used the linear predictors of the logistic regression models using age, gender and each of the markers as regressors [log_10_(MMP-9) or log_10_(FIT + 2)]. The area under the curve (AUC) obtained for each marker were compared using the method of DeLong^35^. Bootstrap (based on B = 2000 resamples) confidence intervals for the difference in AUCs were also provided. Cut-offs for the linear regression model of each marker were determined by fixing specificity to 90.0% or 95.0%. Statistical analyses were accomplished with the SPSS software (v.20.0, SPSS Inc.; Chicago, IL, USA) and R (Wirtschafts Universität, Wien, Austria). All tests were two-sided and *P*-values ≤ 0.05 were considered statistically significant.

## Additional Information

**How to cite this article**: Otero-Estévez, O. *et al.* Serum matrix metalloproteinase-9 in colorectal cancer family-risk population screening. *Sci. Rep.* **5**, 13030; doi: 10.1038/srep13030 (2015).

## Supplementary Material

Supplementary Tables S1 and S2

## Figures and Tables

**Table 1:** Crude serum MMP-9 levels according to demographic and clinical characteristics of the cohort.

Characteristics	N (%)	Mean ± SD (ng/mL)	Median (ng/mL)	Difference in medians (95% CI)^1^	Range (ng/mL)	*p-*value
**Age (years)**						
≤49	179 (34.7)	533 ± 311	451	Ref.	84–2339	<0.001^a^
50–59	173 (33.5)	428 ± 236	376	−75[−138–(−34)]	69–1681	
≥60	164 (31.8)	449 ± 248	402	−49 [−114–(−4)]	31–1965	
**Gender**						
Male	212 (41.1)	512 ± 312	421	Ref.	31–2339	0.013^b^
Female	304 (58.9)	442 ± 234	401	−20 (−86–17)	69–1681	
**Familial risk**						
1 FDR ≥ 60 years	343 (66.5)	460 ± 225	420	Ref.	50–1285	0.652^b^
1 FDR < 60 years or ≥ 2 FDRs	173 (33.5)	476 ± 292	396	−24 (−74–13)	31–2339	

^a^Kruskal-Wallis test.

^b^Mann-Whitney U test; FDR: first-degree relative.

^1^Bootstrap confidence intervals of the median difference of crude MMP-9 among subgroups. The Reference subgroup (that with the largest median) is indicated as Ref.

**Table 2:** Serum MMP-9 levels corrected by age and gender according to the colonoscopy findings.

Colorectal findings	N	Mean ± SD (ng/mL)^1^	Median (ng/mL)^1^	Difference in medians^2^ (95% CI)	Range (ng/mL)^1^	*p*- value^3^
**No neoplasia**	338	642 ± 352	564	Ref.^b^	100–2779	
No colorectal findings	174	633 ± 322	577	Ref.^a^	100–1709	
Benign pathologies	164	653 ± 381	545	−32 (−99–70)^a^	158–2779	0.997^a^
Haemorrhoids	68	651 ± 329	532	−45 (−118–109)^a^	158–1725	0.748^a^
Diverticula	46	683 ± 442	601	24 (−120–137)^a^	200–2482	0.859^a^
No-neoplastic polyps	44	608 ± 403	512	−65 (−132–29)^a^	259–2779	0.328^a^
Other minor findings	6	756 ± 317	601	24 (−26–667)^a^	494–1190	0.271^a^
**Non-advanced adenomas**	121	668 ± 379	599	35 (−61–152)^b^	43–2068	0.579^b^
**Advanced neoplasia**	57	712 ± 356	630	66 (−31–190)^b^	145–2150	0.056^b^
Advanced adenomas	53	722 ± 349	648	84 (−21–196)^b^	179–2150	0.029^b^
Cancer	4	568 ± 474	446	−118 (−435–682)^b^	145–1237	0.452^b^

^1^Values correspond to the MMP-9 corrected by age and gender (cMMP-9).

^2^Bootstrap confidence intervals of the median difference of cMMP-9 among subgroups, considering as Reference (Ref.) the no colorectal findings group^a^ or the no neoplasia group^b^.

^3^Mann-Whitney U test. Comparisons with the no colorectal findings group^a^ and the no neoplasia group^b^.

**Table 3:** Serum MMP-9 levels corrected by age and gender according to the characteristics of adenomas.

Variable	N	Mean ± SD (ng/mL)^1^	Median (ng/mL)^1^	Difference in medians (95% CI)^2^	Range (ng/mL)^1^	*p*-value
**Number**						
1–2	142	676 ± 328	627	Ref.	73–2150	0.567^3^
3 or more	32	721 ± 521	568	59 (−155–198)	43–2068	
**Size**						
<1 cm	127	666 ± 382	586	69 (−47–226)	43–2068	0.072^3^
≥1 cm	47	734 ± 335	655	Ref.	179–2150	
**Histology**						
Tubular	147	667 ± 359	613	78 (−118–232)	43–2068	0.180^3^
Tubulovillous/Villous	27	780 ± 420	691	Ref.	353–2150	
**Location**						
Distal	96	689 ± 357	609	49 (−159–159)	225–2068	
Proximal	41	663 ± 321	658	Ref.	73–1532	0.940^4^
Both	37	696 ± 454	605	53 (−166–188)	43–2150	

^1^Values correspond to the MMP-9 corrected by age and gender (cMMP-9).

^2^Bootstrap confidence intervals of the median difference of cMMP-9 among subgroups, considering as Reference (Ref.) the subgroup with the largest median.

^3^Mann-Whitney U test.

^4^Kruskal-Wallis test.

**Table 4:** Diagnostic performance of MMP-9 and FIT for the detection of advanced neoplasia and advanced adenomas.

Biomarker	Advanced neoplasia			Advanced adenomas		
	Cut-off	Sensitivity (%)	Specificity (%)	Cut-off	Sensitivity (%)	Specificity (%)
MMP-9	0.19	19.30	90	0.18	22.64	90
	0.24	8.77	95	0.23	9.43	95
FIT	0.17	52.63	90	0.16	47.17	90
	0.24	45.61	95	0.22	43.40	95

For MMP-9 (log_10_MMP-9) and FIT [log_10_(FIT + 2)] the confounders gender and age were included in the regression model to assess the diagnostic performance. The cut-offs are based on the regression model of each marker. FIT: Faecal immunochemical test.
