# Supplementary material for: Serum matrix metalloproteinase-9 in colorectal cancer family-risk population screening
Source: Sci Rep. 2015 Aug 12;5:13030. doi: 10.1038/srep13030 (PMC4532998; doi:10.1038/srep13030)
Supplement: Supplementary Tables S1 and S2 [file srep13030-s1.pdf]

Supplementary Information

Title: Serum matrix metalloproteinase 9 in colorectal cancer family-risk population screening

Authors: Olalla Otero-Estévez, Loretta De Chiara, Mar Rodríguez-Girondo, Francisco Javier Rodríguez-Berrocal, Joaquín Cubiella, Inés Castro, Vicent Hernández, Vicenta Soledad Martínez-Zorzano.*

Supplementary Table S1. Gender and age distribution of the individuals from the familial risk cohort according to the colonoscopy findings.

**Colorectal findings**	**Gender**		**Age**		
(number of cases)	Male	Female	≤49	50-59	≥60
**No neoplasia** (338)	120 (35.5%)	218 (64.5%)	143 (42.3%)	102 (30.2%)	93 (27.5%)
No colorectal findings (174)	63 (36.2%)	111 (63.8%)	90 (51.7%)	60 (34.5%)	24 (13.8%)
Benign pathologies (164)	57 (34.8%)	107 (65.2%)	53 (32.3%)	42 (25.6%)	69 (42.1)
Haemorrhoids (68)	23 (33.8%)	45 (66.2%)	33 (48.5%)	15 (22.1%)	20 (29.4%)
Diverticula (46)	13 (28.3%)	33 (71.7%)	5 (10.9%)	10 (21.7%)	31 (67.4%)
No-neoplastic polyps (44)	20 (45.5%)	24 (54.5%)	12 (27.3%)	15 (34.1%)	17 (38.6%)
Other minor findings (6)	1 (16.7%)	5 (83.3)	3 (50.0%)	2 (33.3%)	1 (16.7%)
**Non-advanced adenomas** (121)	57 (47.1%)	64 (52.9%)	26 (21.5%)	44 (36.4%)	51 (42.1%)
**Advanced neoplasia** (57)	35 (61.4%)	22 (38.6%)	10 (17.5%)	27 (47.4%)	20 (35.1%)
Advanced adenomas (53)	32 (60.4%)	21 (39.6%)	9 (17.0%)	24 (45.3%)	20 (37.7%)
Colorectal cancer (4)	3 (75.0%)	1 (25.0%)	1 (25.0%)	3 (75.0%)	0 (0%)

Supplementary Table S2. Crude serum MMP-9 levels according to the colonoscopy findings.

**Colorectal findings**	**N**	**Mean ± SD (ng/mL)**	**Median (ng/mL)**	**Range (ng/mL)**
**No neoplasia**	338	462.68 ± 264.44	398.47	68.46-2338.93
No colorectal findings	174	465.80 ± 250.19	406.13	68.46-1537.16
Benign pathologies	164	459.37 ±29.49	385.29	104.06-2338.93
Haemorrhoids	68	471.20 ± 235.14	414.32	104.06-1132.13
Diverticula	46	449.94 ± 286.67	402.02	132.95-1681.38
No-neoplastic polyps	44	441.47 ±345.09	355.75	181.37-2338.93
Other minor findings	6	528.74 ± 175.08	475.23	357.42-740.41
**Non-advanced adenomas**	121	472.74 ± 284.27	430.32	30.97-1964.83
**Advanced neoplasia**	57	515.34 ± 280.91	426.72	97.72-1836.37
Advanced adenomas	53	520.89 ± 273.67	438.78	119.16-1836.37
Cancer	4	441.83 ± 408.78	319.94	97.72-1029.73
